# Outcomes of low-risk birth care during the Covid-19 pandemic: A cohort study from a tertiary care center in Lithuania

**DOI:** 10.1515/med-2023-0720

**Published:** 2023-05-27

**Authors:** Ingrida Poškienė, Meilė Minkauskienė, Rima Kregždytė, Kristina Jarienė, Mindaugas Kliučinskas

**Affiliations:** Department of Obstetrics and Gynaecology, Medical Academy, Lithuanian University of Health Sciences, Kaunas, Lithuania; Department of Preventive Medicine, Medical Academy, Lithuanian University of Health Sciences, Kaunas, Lithuania

**Keywords:** midwife, low-risk birth, Covid-19 infection, birth outcome

## Abstract

According to the World Health Organization, midwife-led care is the most appropriate and cost-effective type of perinatal care. As the Covid-19 pandemic with its drastic changes and challenges for the health systems and the medical staff made large adjustments to the healthcare delivery system, midwife-led care became an even more important supportive tool in maintaining unnecessary interventions. This retrospective cohort study aims to compare the outcomes of midwife-led care and team-led care in low-risk births between the Covid-19 pandemic and non-Covid-19 pandemic period. The total studied population was 1,185 singleton births and consisted of 727 births during the non-Covid-19 period and 458 births from the Covid-19 period. The study revealed the safety of low-risk birth care during the first wave of the Covid-19 pandemic in both groups. The maternal and perinatal outcomes remained stable without an increased rate of unsuccessful vaginal births and newborn asphyxia; moreover, birth care of low-risk women provided by midwives preserved autonomy, integrity, and resistance to responding to a disaster. The aforementioned results exhibit that high-quality, safe supervision by midwives in low-risk births can be provided even in high-stress circumstances.

## 1 Introduction

The term “midwife” is derived from the Old English word mid, “with,” and wif, “woman,” and thus originally meant the person who is with the woman (mother) at childbirth. In many countries, midwives are the primary caretakers, leading and supervising maternity care from the initial booking to the postnatal period [1]. According to the World Health Organization, midwife-led care is the most appropriate and cost-effective type of perinatal care [2]. It is well documented that midwife-led care is imperative to improving the quality of care and outcomes, and being a more efficient use of health care resources by reducing the rates of maternal and neonatal mortality and morbidity, stillbirths, and preterm births, as well as decreasing the number of interventions and unnecessary medicalizations, and improving the psychosocial and public health outcomes [3]. Midwife-led care consists of continuous care and monitoring of the physical, psychological, spiritual, and social well-being of women throughout the childbearing cycle [4,5]. Supportive, technically competent midwives, emotional support, and a psychologically safe environment are crucial for a positive childbirth experience. Usually, the midwife provides care to healthy women with low-risk pregnancies, recognizes risks, and thus, continues to give midwifery care (MC) alongside a team of other professionals to women who experience medical and obstetric complications.

For almost half of the century, Lithuania had an obstetrician–gynecologist-led maternity care system, in which midwives were a part of the system without any autonomy in their practice. Midwives were attenuated to the position of doctor’s assistant and had very limited responsibilities. The recent decades prompted tremendous changes toward enhancing the autonomy of midwives. Nowadays, midwives independently provide antenatal and birth care for low-risk women in Lithuania for about 5% of births. Currently, there are two main models of care in Lithuania: midwife-led and physician-led care for the perinatal period. The former model, i.e., the midwife-led care model, is based on a normal physiological process of birth, focusing on personalized care and avoiding unnecessary medical interventions. The latter model, physician-led care, is focused on safety aspects at birth. Therefore, low-risk pregnant women can choose either midwife- or physician-led care. However, in some cases, low-risk pregnancy can switch to high risk – shifting from the normal process of birth to the need for medical interventions. In such cases, the obstetrician–gynecologist will also be involved in the care process and medical staff will work as a team.

The COVID-19 pandemic caused a plethora of challenges and had a tremendous impact on medical practices, including maternity care. The pandemic caused many disruptions and modifications to the maternity services, such as social distancing between the midwife/doctor and the patient during delivery, which could impact even the perinatal outcomes of low-risk healthy women groups [6].

The aim of this study was to investigate the effects of the first wave of the Covid-19 pandemic on maternal and neonatal outcomes in low-risk deliveries.

## 2 Methods

### 2.1 Study design

Retrospective cohort study.

### 2.2 Research question

Did low-risk birth care remain safe and skilled in the Covid-19 pandemic? Did the medical and communication restrictions implemented due to Covid-19 alter the low-risk birth results?

### 2.3 Setting

This study was performed in The Hospital of the Lithuanian University of Health Sciences (LSMU), Kauno klinikos, which is the largest academic healthcare institution in Lithuania. The Hospital of LSMU is Perinatology Center that provides tertiary care to approximately 3,000 deliveries per year. This institution also caters to deliveries of the highest risk, although it has the biggest proportion of low-risk deliveries in Lithuania (which are autonomously supervised by certified midwives).

### 2.4 Study participants

This research studied low-risk women with singleton pregnancies, who were supervised by midwives and gave vaginal birth beyond the 37 weeks of gestation. The Covid-19 period of study lasted from March 16, 2020 until March 15, 2021. The Covid-19 pandemic in Lithuania was announced on March 14, 2020. For this study, the first year of the pandemic has been selected (and henceforth referred to) as the pandemic period, which had the strictest national restrictions. The period between March 16, 2018 and March 15, 2019 was defined as the non-Covid-19 period, and women who gave birth during this period were considered as controls.

A low-risk birth generally is deemed if a healthy woman had a pregnancy without any complications, and her fetus was mature and healthy (beyond 37 weeks of gestation). For a low-risk birth, midwife-led care generally is proposed at first; however, if the pregnant woman does not agree, an option of physician-led care is also available. If the risk during labor changes to medium or high, the obstetrician–gynecologist also is involved in the care process and medical staff will work as a team.

Two study subgroups were conducted:

1) A low-risk birth with MC.
2) A low-risk birth with team care (TC), i.e., the risk has increased during labor, and hence, the obstetricians–gynecologists were involved in care along with midwives.

All patients were included in the study period, regardless of if the SARS-CoV-2 status was later confirmed as positive. Women having any Covid-19 symptoms were categorized as not low-risk parturients; thus, only asymptomatic cases were possibly included in the study group.

### 2.5 Study size

The total studied population was 1,185 singleton births, consisting of 727 births during the non-Covid-19 period and 458 births from the Covid-19 period.

### 2.6 Variables

Data were collected using the hospital’s electronic database. Sociodemographic variables, such as age, urban or rural living, education, marital status, gravidity and parity, and gestational age in weeks, were obtained from medical records. Data on labor and neonatal outcome variables included the following: uterine revision, instrumental labor, perineal tears, birth induction of labor, pain relief methods, postpartum hemorrhage, hospital stay duration (in days), newborn weight, length, gender, and Apgar score at 1 and 5 min.

Successful spontaneous vaginal birth (i.e., normal birth) was categorized as a labor that started spontaneously, progressed spontaneously, and had given childbirth spontaneously without any intervention.

### 2.7 Data analysis and statistical methods

Statistical analysis was performed using IBM SPSS Statistics 27 (IBM Corp., Armonk, NY, USA). Descriptive statistics of quantitative and qualitative variables was calculated. The mean, median, standard deviation, interquartile range, minimum, and maximum values were calculated for quantitative variables. Percentages were calculated for qualitative variables. Quantitative data were tested for distribution normality using the Shapiro–Wilk test. The parametric *t*-test or the nonparametric Mann–Whitney *U* test was applied to compare quantitative variables between two independent groups. The Chi-square test or the Fisher–Freeman–Halton exact test was applied to compare qualitative variables between independent groups. Differences were considered statistically significant if *p* < 0.05.


**Ethical and data protection considerations:** The study protocol was reviewed and approved by the Biomedical Research Ethics Committee of the Kaunas region (No. BE-2-33). There was no direct contact with births and neonates during data collection and analysis. No maternal and neonatal personally identifiable information was included in the research data; therefore, the Biomedical Research Ethics Committee of Kaunas region exempted the informed consent form usage.

## 3 Results

**Figure 1:**
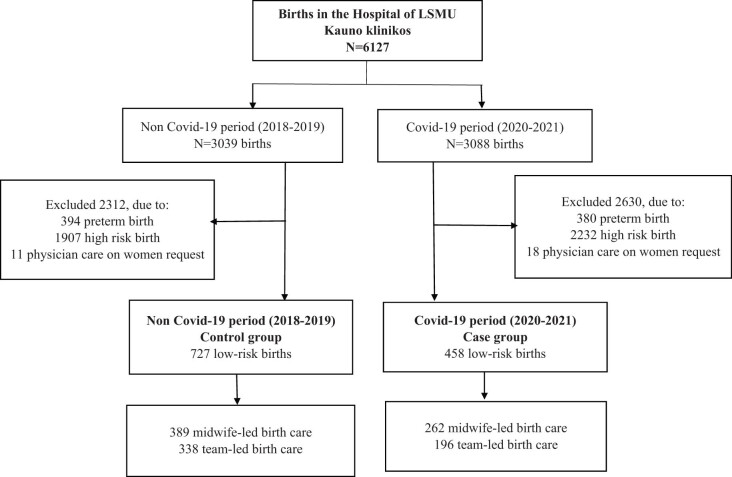
Study flowchart.

There were 3,088 deliveries registered during the first wave of the COVID-19 pandemic (2020–2021) at the study hospital. About 3,030 deliveries were conducted in the control group (2018–2019). Figure 1 presents 1,185 low-risk births that fulfill inclusion criteria and were analyzed in Covid-19 and non-Covid-19 groups.

Table 1 summarizes the sociodemographic and clinical characteristics of the study and control groups. The low-risk women from the Covid-19 group were, on average, older compared with that in the non-Covid-19 group (30.6 ± 5.3 vs 29.5 ± 4.9 years, *p* < 0.001). Maternal education, place of living, marital status, gravidity, and parity were all similar between groups. The nulliparity rate did not differ between groups (44.1 vs 42.1%, *p* = 0.745). When comparing descriptive characteristics, such as allergies (6.8 vs 3.2%, *p* = 0.004), cardiovascular diseases (2.2 vs 0.3%, *p* = 0.001), and urinary-tract diseases in pregnancy (3.9 vs 0.6%, *p* < 0.001), they were statistically significant more commonly in the study group compared to the control group. The rate of gestational hypertensive disorders and gestational diabetes did not differ between groups. Gestational anemia was more prevalent (4.1 vs 10.7%, *p* < 0.001) in the non-Covid-19 group. The mean gestational age presented no difference between the study groups.

**Table 1:** Sociodemographic and clinical characteristics of the study and control group

Variable	COVID-19 period (*n* = 458)	Non-COVID-19 period (*n* = 727)	*p* value
Maternal age (years) (mean, SD)	30.6 (5.3)	29.5 (4.9)	**<0.001**
Maternal education			
Primary/basic	18 (3.9)	21 (2.9)	0.598
Secondary/upper	167 (36.5)	250 (34.5)	
University	273 (59.6)	456 (62.6)	
Marital status			
Single	89 (19.4)	128 (17.6)	0.429
Living with partners	369 (80.6)	599 (82.4)	
Parity (mean, SD)	1.66 (0.7)	1.67 (0.7)	0.835
Gravidity (mean, SD)	1.87 (0.9)	1.93 (1.1)	0.685
Gestational age (weeks, mean, SD)	39.4 (1.0)	39.4 (1.0)	0.306
Living place			
Urban	304 (66.4)	482 (66.3)	0.900
Rural	154 (33.6)	245 (33.7)	
Maternal diseases			
Allergy	31 (6.8)	23 (3.2)	**0.004**
Cardiovascular diseases	10 (2.2)	2 (0.3)	**0.001**
Urinary-tract diseases	18 (3.9)	4 (0.6)	**<0.001**
Maternal hypertension disorder	4 (0.9)	2 (0.3)	0.158
Gestational anemia	19 (4.1)	78 (10.7)	**<0.001**
Gestational diabetes	12 (2.6)	20 (2.8)	0.892

Note: Continuous variables were reported as mean ± SD and categorical variables were reported as counts and percentages. *p*-values were determined by the Mann–Whitney *U* test for continuous variables and the Chi-square test for categorical and binary variables. *T-*test was applied to birthweight.

Bold represents statistically significant, *p* < 0.05.

Labor outcomes of low-risk deliveries during or before the first wave of the Covid-19 pandemic are presented in Table 2.

**Table 2:** Labor outcomes of low-risk deliveries during or prior the first wave of Covid-19 pandemic

Variable	Midwife (*N* = 651)			Team (*N* = 534)			COVID-19 period (*n* = 458)	Non-COVID-19 period (*n* = 727)	*p* value
	COVID-19 period (*n* = 262)	Non-COVID-19 period (*n* = 389)	*p* value	COVID-19 period (*n* = 196)	Non-COVID-19 period (*n* = 338)	*p* value			
Birth induction									
None	217 (82.8)	313 (80.5)	0.138	71 (36.2)	143 (42.3)	0.328	288 (62.9)	456 (62.7)	0.408
Amniotomy	39 (14.9)	52 (13.4)		30 (15.3)	46 (13.6)		69 (15.1)	98 (13.5)	
Oxytocinum	4 (1.5)	15 (3.9)		72 (36.7)	102 (30.2)		76 (16.6)	117 (16.1)	
Amniotomy + oxytocinum	2 (0.8)	9 (2.2)		19 (9.7)	43 (12.7)		21 (4.6)	52 (7.2)	
Misoprostolum	0 (0.0)	0 (0.0)		4 (2.1)	4 (1.2)		4 (0.8)	4 (0.5)	
Delivery analgesia									
None	88 (33.6)	214 (55.0)	**<0.001**	43 (21.9)	150 (44.4)	**<0.001**	131 (28.6)	364 (50.1)	**<0.001**
NO_2_	69 (26.3)	88 (22.6)		47 (24.0)	64 (18.9)		116 (25.3)	152 (20.9)	
Spinal-epidural	37 (14.1)	69 (17.7)		86 (43.9)	122 (36.1)		123 (26.9)	191 (26.3)	
General i/v	3 (1.1)	0 (0.0)		2 (1.0)	0 (0.0)		5 (1.1)	0 (0.0)	
Hydrotherapy	22 (8.5)	15 (3.9)		3 (1.5)	0 (0.0)		25 (5.5)	15 (2.1)	
Other non-medications	43 (16.4)	3 (0.8)		15 (7.7)	2 (0.6)		58 (12.6)	5 (0.6)	
Perineal tears									
None	70 (26.7)	99 (25.4)	0.774	48 (24.5)	90 (26.6)	0.411	118 (25.8)	189 (26.0)	0.700
Episiotomy	68 (26.0)	108 (27.8)		85 (43.4)	128 (37.9)		153 (33.4)	236 (32.5)	
First- and second-degree tears	123 (46.9)	182 (46.8)		62 (31.6)	119 (35.2)		185 (40.4)	301 (41.4)	
Third- and fourth-degree tears	1 (0.4)	0 (0.0)		1 (0.5)	1 (0.3)		2 (0.4)	1 (0.1)	
Revision	0 (0.0)	0 (0.0)	**-**	8 (4.1)	11 (3.3)	0.373	8 (1.8)	11 (1.5)	0.500
Instrumental	0 (0.0)	0 (0.0)	**-**	7 (3.6)	6 (1.8)	0.373	7 (1.5)	6 (0.8)	0.500
Cesarean section	—	—	**-**	17 (8.6)	35 (10.3)	0.528	17 (3.7)	35 (4.8)	0.528
Blood loss after vaginal birth >500 ml	1 (0.4)	0 (0.0)	0.402	12 (6.7)	19 (6.3)	0.851	13 (2.9)	19 (2.7)	0.841
Successful vaginal birth	69 (26.3)	89 (22.9)	0.313	16 (8.2)	34 (10.1)	0.469	85 (18.6)	123 (16.9)	0.470
Unsuccessful vaginal birth	193 (73.7)	300 (77.1)	0.351	163 (83.2)	269 (79.6)	0.539	356 (77.7)	569 (78.3)	0.470
Postpartum hospitalization (days, SD)	2.9 (1.3)	3.5 (1.6)	**<0.001**	3.6 (1.7)	3.5 (1.4)	1.000	3.2 (1.5)	3.5 (1.5)	**<0.001**

Note: Continuous variables were reported as mean ± SD and categorical variables were reported as counts and percentages. *p*-values were determined by the Mann–Whitney *U* test for continuous variables and the Chi-square test for categorical and binary variables.

Bold represents statistically significant, *p* < 0.05.

The rate of labor induction did not differ in the low-risk parturient of Covid-19 and non-Covid-19 groups (37.1 vs 37.3%, *p* = 0.956). As anticipated, the labor induction rate in the midwife-led care subgroup was even less common than in the common group in Covid-19 and non-Covid-19 periods (82.8 vs 80.5%), mostly used by performing amniotomy. Furthermore, the team-led care of low-risk women had higher rates of oxytocin and misoprostol use for labor induction compared to midwife-led care, without a difference in Covid-19 and non-Covid-19 periods (36.7 vs 1.5% and 30.2 vs 3.9%, 2.1 vs 0% and 1.2 vs 0% respectively, *p* < 0.001).

The rate of low-risk women who gave birth without any perineal tears was similar in all analyzed groups, as about one-quarter of women delivered with intact perineum in a midwife- and team-led groups, during Covid-19 and non-Covid-19 periods (26.7 vs 24.5% and 25.4 vs 26.6%, respectively). However, in the midwife-led group, first- and second-degree tears were most prevalent (46.9 and 46.8% in Covid-19 and non-Covid-19 periods, respectively) as episiotomy were performed less frequently (26.0 and 27.8% in Covid-19 and non-Covid-19 periods, respectively). On the contrary, the team-led group had chosen to perform episiotomy more often compared to low-grade spontaneous perineal tears in both non-Covid-19 and Covid-19 periods (37.9 and 35.2% for episiotomy vs 43.4 and 31.6% for low-grade tears in both periods, *p* = 0.411). The occurrence of high-grade perineal tears was extremely rare in all analyzed groups, showing no difference in Covid-19 and non-Covid-19 periods.

Analysis of analgesia during labor showed that women in the Covid-19 group used any analgesia more often compared to the non-Covid-19 group in midwife-led (66.4 vs 45%, *p* < 0.001), team-led (78.1 vs 55.6%, *p* < 0.001), and common groups (71.4 vs 49.9%, *p* < 0.001). The rates of epidural analgesia were similar in Covid-19 and non-Covid-19 groups (26.9 vs 26.3%) for all low-risk women. Nevertheless, women in the Covid-19 group were more likely to choose hydrotherapy (5.5 vs 2.1%, *p* < 0.001) and other nonpharmacological analgesia (12.6 vs 0.6%, *p* < 0.001) compared to the non-Covid-19 group, showing similar differences in midwife-led and team-led subgroups.

The proportion of successful vaginal births did not differ in joint low-risk birth care between the Covid-19 and non-Covid-19 groups (18.6 vs 16.9%, *p* = 0.470). Comparing vaginal birth success rates in the subgroups showed some insignificant differences, revealing that the Covid-19 period had slightly higher rates of successful vaginal births in the midwife-led group, while the team-led group showed less success. Analysis of successful vaginal births in the midwife-led care subgroup showed that the Covid-19 period had a statistically insignificant higher rate (26.3 vs 22.9%, *p* = 0.313) of successful vaginal births compared to the non-Covid-19 period. Furthermore, the Covid-19 group with team-led care demonstrated a statistically insignificant decrease in successful vaginal birth rate compared to the non-Covid-19 team-led care group (8.2 vs 10.1%, *p* = 0.469).

Instrumental delivery, uterine revision, and blood loss after vaginal birth (>500 ml) were uncommon in midwife-led and team-led groups in both Covid-19 and non-Covid-19 periods and thus demonstrated no significant difference. The birth was ended by cesarean section in 3.7% of low-risk parturients in the Covid-19 group and 4.8% in the low-risk parturient in the non-Covid-19 group, suggesting no significant difference in the occurrence of vaginal births (*p* = 0.528) between periods.

The mean length of postpartum hospitalization was significantly shorter during the Covid-19 period than during the non-Covid-19 period in midwifery-led (2.9 ± 1.3 vs 3.5 ± 1.6, *p* < 0.001) and common groups (3.2 ± 1.5 vs 3.5 ± 1.5, *p* < 0.001). There was no difference in the postpartum length of stay between the Covid-19 period and the non-Covid-19 period in the team-led group (3.6 ± 1.7 vs 3.5 ± 1.4, *p* = 1.000).

The neonatal outcomes are depicted in Table 3. The comparison of newborn data showed no difference in the birth weight, although newborns in the Covid-19 group were, on average, 3 cm taller compared to the non-Covid-19 group (52.4 vs 52.1 cm, *p* < 0.001). The rates of newborns with an Apgar score below ≤7 after 1 and 5 min, as well as their mean Apgar scores, had no significant difference when compared with the Covid-19 period and non-Covid-19 period in midwife-led, team-led, and common groups.

**Table 3:** Neonatal outcomes of low-risk deliveries during or before the first wave of Covid-19 pandemic

Outcomes	Midwife (*N* = 651)			Team (*N* = 534)			COVID-19 period (*n* = 458)	Non-COVID-19 period (*n* = 727)	*p* value
	COVID-19 period (*n* = 262)	Non-COVID-19 period (*n* = 389)	*p* value	COVID-19 period (*n* = 196)	Non-COVID-19 period (*n* = 338)	*p* value			
Apgar score									
Apgar 1 min ≤7	1 (0.4)	0 (0.0.)	0.403	12 (6.1)	10 (3.0)	0.112	13 (2.8)	10 (1.4)	0.403
Apgar 5 min ≤7	1 (0.4)	0 (0.0)	0.402	3 (1.5)	2 (0.6)	0.362	4 (0.9)	2 (0.3)	0.402
Apgar 1 min score	9.5 (0.6)	9.5 (0.6)	0.653	9.1 (1.2)	9.1 (0.9)	0.492	9.3 (0.9)	9.3 (0.8)	0.265
Apgar 5 min score	9.8 (0.4)	9.9 (0.3)	0.606	9.7 (0.7)	9.7 (0.6)	0.441	9.8 (0.6)	9.8 (0.5)	0.248
Birthweight (g)	3501.8 (368.3)	3523.4 (416.0)	0.460	3542.0 (419.2)	3523.9 (414.0)	0.628	3519.0 (390.9)	3523.7 (414.8)	0.812
Birthweight ≤2,500 g	1 (0.4)	1 (0.3)	0.724	2 (1.0)	2 (0.6)	0.559	3 (0.7)	3 (0.4)	0.748
Birthweight ≥4,500 g	1 (0.4)	1 (0.3)	0.724	1 (0.5)	5 (1.5)	0.559	2 (0.4)	6 (0.8)	0.748
Birth height (cm)	52.3 (2.4)	52.0 (2.3)	**0.022**	52.5 (2.2)	52.2 (2.3)	**0.019**	52.4 (2.3)	52.1 (2.3)	**<0.001**
Neonatal gender									
Male	118 (45.0)	191 (49.1)	0.309	96 (49.0)	175 (51.8)	0.533	214 (46.7)	366 (50.3)	0.225
Female	144 (55.0)	198 (50.9)		100 (51.0)	163 (48.2)		244 (53.3)	361 (49.7)	

Note: Continuous variables were reported as mean ± SD and categorical variables were reported as counts and percentages. *p*-values were determined by the Mann–Whitney *U* test for continuous variables and the Chi-square test for categorical and binary variables.

Bold represents statistically significant, *p* < 0.05.

## 4 Discussion

### 4.1 Key results and their interpretation

This study aimed to investigate the effects of the first wave of the Covid-19 pandemic on birth care in low-risk deliveries. The hospital selected for this study had similar Covid-19 restrictions to other hospitals in various countries during the pandemic. Lockdown restrictions have changed natural birth care completely, implementing strict control and extraordinarily terrifying regulations. Moreover, it was hypothesized that these adjustments might lead to poorer birth outcomes. After the Covid-19 virus was categorized as a pandemic, the Ministry of Health and Lithuanian Society of Obstetricians of Gynecologists issued guidelines for safe healthcare provision and delivery of obstetric care during the Covid-19 era. These guidelines addressed the strict use of personal protective equipment in hospitals, including during the pushing stage of labor. Mandatory Covid-19 testing for all hospital admissions was introduced. Antepartum or postpartum visitors were prohibited. For the first 3 months of lockdown, the attendance of any support person in the delivery room was forbidden. Later, only one support person was allowed in the delivery room. The social distancing between parturient and midwife or other medical staff was strongly encouraged until a negative Covid-19 test was received; any physical contact was restricted only to necessary interventions. The medical staff waiting for a negative Covid-19 test used the highest level of protective equipment. The number of people in a delivery room was reduced to a minimal amount. The aforementioned modifications and restrictions had a major psychological impact on delivering women’s well-being. Anxiety, fear, loneliness, and depression were more common in pregnant women during the pandemic period [7,8]. Negative emotional well-being was extremely devastating for the woman during the birth when pain, staying alone, and fear of not receiving support on time would create a negative childbirth experience [9,10]. On the contrary, some studies demonstrate that despite the extreme conditions and dramatic changes in birth care during the Covid-19 pandemic, midwives were qualified and prepared to provide delivery service [11].

The National data from Austria showed significantly increased rates of labor induction, instrumental delivery, NICU transfer, low Apgar scores, and postpartum adverse events during the Covid-19 period [12]. Results from a retrospective cohort (that was conducted in a tertiary medical center in New York City) have indicated that significantly higher rates of hypertensive disorders of pregnancy, medically indicated preterm birth, and PPH were seen even in the absence of SARS-CoV2 infection during the pandemic period [13]. A systematic review with a meta-analysis from 38 studies revealed that Covid-19 restrictions had no overall effect on preterm birth at less than 37 weeks, a significant reduction in preterm births at less than 34 weeks, no effect on the risk of stillbirth or birth weight, and significantly reduced NICU admission rates [14]. This study has analyzed exceptionally low-risk deliveries, where care was provided by midwives or teams in hospitals. Home deliveries are legalized but are extremely rare in Lithuania, and women’s choice to give birth in the hospital did not change during the Covid-19 pandemic. The study findings suggest that rates of successful vaginal births, neonatal outcomes, and major obstetrical complications were similar to the non-pandemic period for low-risk deliveries. Furthermore, cesarean sections and perineal tear rates showed no difference between the studied periods. Despite the increased need for any analgesia in labor, the use of epidural analgesia remained stable, and of higher necessity in nonpharmacological methods, nitric oxide analgesia, or other intravenous medications were noticed. These findings are partially in line with other studies, showing the shorter duration of hospital stay during the Covid-19 pandemic [12,15]. The differences in the rates of maternal diseases between periods are not easy to explain. They might be associated with increased fear and reporting, or less attendance to antenatal visits during the lockdown.

### 4.2 Strengths and weaknesses

This study is noteworthy as it analyzes low-risk deliveries during the Covid-19 pandemic. Despite strict regimens and tremendous changes in the hospital's organizational and communicational structure during the lockdown, the study confirms that low-risk births can be supervised by midwives safely and with high quality.

The results of this study not only allow midwives to have confidence in their abilities as health care professionals to provide high-quality care during stressful circumstances but as well portrays that obstetricians–gynecologists and patients should feel secure in midwife care at any time. The study results allow for more efficient use of human resources in providing obstetric care to low- or high-risk birth.

The strength of the study is that strict inclusion and exclusion criteria were used. The data from one perinatal center are presented with uniform data documentation and standardized clinical management, having the biggest practice of midwife-led care of low-risk women in the country. The following study on midwife-led care during the Covid-19 pandemic is the first research investigating the impact of pandemic restrictions on low-risk births and delivery care. A single-center study potentially misses the changes at different hospitals with different restrictions. For any future research, the analysis of national data (including all low-risk midwife-led deliveries) would help to obtain more reliable and robust results. The changes in antenatal care (variables such as the number of follow-up appointments during pregnancy, the precocity of the pregnant woman’s first appointment, and missed consultations) that are related to birth success were not included in the analysis due to the retrospective design of the study. Some differences in the rates of maternal diseases can be explained by the lower accessibility of some out-patient visits.

This study has several other limitations. It is a retrospective study with a limited size of control and study groups, which precludes the analysis of rare maternal and newborn complications. Due to the design of this study, we are unable to explain some correlations. The short-term outcomes were analyzed; on the other hand, long-term maternal and neonatal outcomes should be of great interest to any future research.

All delivering women were tested for Covid-19, and positive results were accounted as a high-risk group. However, it is still possible that some low-risk women delivered while being Covid-19 positive, and this also might have influenced the results.

## 5 Conclusion

The study revealed the safety of low-risk birth care during the first wave of the Covid-19 pandemic. Despite big changes in the policy in the labor and delivery unit, the maternal and perinatal outcomes remained stable without any increase in the rate of unsuccessful vaginal birth and newborn asphyxia. Although the maternal length of stay in the hospital was significantly shorter, birth care of low-risk women provided by midwives preserved autonomy, integrity, and resistance to respond to disaster.
